# Puerarin ameliorated the behavioral deficits induced by chronic stress in rats

**DOI:** 10.1038/s41598-017-06552-x

**Published:** 2017-07-24

**Authors:** Zhi-Kun Qiu, Guan-Hua Zhang, De-Sheng Zhong, Jia-Li He, Xu Liu, Ji-Sheng Chen, Da-Nian Wei

**Affiliations:** 1 Pharmaceutical Department of The First Affiliated Hospital of Guangdong Pharmaceutical University, Clinical Pharmacy Department of Guangdong Pharmaceutical University, Guangzhou, 510080 P.R. China; 2 Neurosurgery Department of the Third Affiliated Hospital of Southern Medical University, Guangzhou, 510630 P.R. China; 3 Department of Pharmacy, Hui Zhou Municipal Centre Hospital, Huizhou, Guangdong P.R. China; 4 Department of Endocrinology, Guangdong Provincial Hospital of Chinese Medicine, Guangzhou, 510120 P.R. China; 5 Pharmacy Department of General Hospital of Chinese People’s Armed Police Forces, Beijing, 100039 P.R. China; 6 Guangdong Provincial Key Laboratory of Pharmacodynamic Constituents of TCM and New Drugs Research, College of Pharmacy, Jinan University, Guangzhou, 510632 P.R. China; 7 Academy of Military Medical Sciences, Beijing, 100850 P.R. China

## Abstract

The present study aimed to investigate the mechanisms underlying the antidepressant-like effects of puerarin via the chronic unpredictable stress (CUS) procedure in rats. Similar to Sertraline (Ser), Chronic treatment of puerarin (60 and 120 mg/kg, i.g) elicited the antidepressant-like effects by reversing the decreased sucrose preference in sucrose preference test (SPT), by blocking the increased latency to feed in novelty-suppressed feeding test (NSFT) and the increased immobility time in forced swimming test (FST) without affecting locomotor activity. However, acute puerarin treatment did not ameliorate the antidepressant- and anxiolytic- like effects in FST and NSFT, respectively. In addition, enzyme linked immunosorbent assay (ELISA) and high performance liquid chromatography-electrochemical detection (HPLC-ECD) showed that chronic treatment of puerarin (60 and 120 mg/kg, i.g) reversed the decreased levels of progesterone, allopregnanolone, serotonin (5-HT) and 5-Hydroxyindoleacetic acid (5-HIAA) in prefrontal cortex and hippocampus of post-CUS rats. Furthermore, puerarin (60 and 120 mg/kg, i.g) blocked the increased corticotropin releasing hormone (CRH), corticosterone (Cort) and adrenocorticotropic hormone (ACTH). Collectively, repeated administration of puerarin alleviated the behavioral deficits induced by chronic stress which was associated with the biosynthesis of neurosteroids, normalization of serotonergic system and preventing HPA axis dysfunction.

## Introduction

Major depressive disorder (MDD) is a debilitating and chronic disorder with high probability of medical and psychiatric co-morbidity, functional impairment, as well as significant societal and personal costs^1, 2^. Although several novel drug targets have been identified, there are no major breakthroughs in the treatment of the disorder^3^. In view of the pathways, a number of drugs are considered beneficial to combat depressive-like behavior. For instance, selective serotonin reuptake inhibitors (SSRIs), such as sertraline (Ser) and paroxetine, are the usual treatment options^4^. Nonetheless, long-term use of these drugs induces multiple inevitable side effects, including cognitive dysfunction, sexual dysfunction, dependence, weight gain, sedation and withdrawal^5, 6^. Based on these drawbacks, considerable effort has been invested in search for better drugs for more effective treatments of depressive-like behavior.

There has been considerable popular interest in using natural extracts and plant preparations to treat depressive-like disorder^7^. Puerarin is a flavonoid glycoside that is extracted from the root of the leguminous plants pueraria lobata and Thomson Kudzuvine Root, and its chemical name is 8-β-D-glucopyranosyl-4′,7-dihydroxyisoflavone^8, 9^. Puerarin displays a series of beneficial activities on cardiovascular disease, hangover, osteoporosis, fever, and liver injury^10–14^. In neurological study, puerarin exerts the potential effects on attenuating memory and learning disorders^15^. Moreover, depressive-like behaviors and chronic pain with spared nerve injury (SNI) in mice are ameliorated by puerarin^16^. However, the molecular and cellular mechanisms underlying the antidepressant-like effects of puerarin are remain unclear.

A greater understanding of the mechanisms underlying depressive-like illness is required to provide new perspectives on the cause and the potential identification of novel therapeutic targets to treat depressive-like behavior. Chronic stress results in the dysregulation of hypothalamic-pituitary-adrenal (HPA) axis, which maybe one of the factors to MDD^17, 18^. The HPA axis, includes a feedback loop composed of the hypothalamus, pituitary and adrenal glands. Hyperactivity of the HPA axis in stress/depressive-like behavior is thought to be particularly related to reduced feedback inhibition by endogenous hormones of corticotropin releasing hormone (CRH), corticosterone (Cort) and adrenocorticotropic hormone (ACTH), which are commonly detected hormones in clinical patients and animal models^19–21^.

In addition, evidences also suggested that abnormal monoaminergic neurotransmission is one of most important mechanisms underlying depressive-like behavior. Numerous studies demonstrated that monoamines were important neurotransmitters involved in the etiology of depressive-like behavior^3, 4^. Actually, most of the antidepressants act on more than one mechanisms, such as inhibition of the reuptake of serotonin (5-HT) and its metabolites. Evidences from various studies indicated that the levels of monoamine neurotransmitters in the brain were increased after the treatments of antidepressants^22^.

Moreover, the down-regulation of neurosteroid biosynthesis has been implicated as one of the possible contributors to the development of depressive disorders^23, 24^. Neuroactive steroids (e.g progesterone and allopregnanolone) have been shown to elicit antidepressant-like properties. For instance, normalization of cerebrocortical allopregnanolone levels may contribute to the pharmacological profile of the antidepressants (i.e SSRIs) in rats^25, 26^.

Based on the above findings, we used a chronic unpredictable stress (CUS) procedure of rats, a well validated stress-related animal model of depressive-like behavior to further evaluate whether puerarin attenuates the CUS behavioral deficits in various behavioral tests. To investigate the molecular and cellular mechanisms underlying the effects of puerarin, we then assessed biosynthesis of neurosteroids production, HPA axis activation and the levels of monoamines in post-CUS rats after chronic puerarin treatment.

## Materials and Methods

### Drugs and reagents

Both puerarin and sertraline (Ser) were purchased from Sigma-Aldrich (St Louis, MO, U.S.A.), dissolved in dimethyl sulphoxide (DMSO, <0.1%) and diluted to indicate concentration with 0.9% normal saline. All compounds were administered by intragastric gavage (i.g.) in a volume of 2 mL/kg between 08:00 and 09:00 h. Ser was administrated as a positive control in all behavioral tests at a dose (15 mg/kg i.g) based on previous PTSD studies^27, 28^. Doses of puerarin (30, 60 and 120 mg/kg i.g) were based on a previous study showing that puerarin ameliorated the depressive-like behavior in rodents^16^.

### Animals

The Sprague-Dawley rats (male, 200 ± 20 g) were maintained in the conditions of controlled humidity (45–50%), temperature (23 ± 1 °C), and lighting (12 h/d). The rats were housed in a 12-h light/dark cycle starting at least 5 days before the experiments with access to food and water freely available. The experiments were performed according to the National Institute of Health Guide for the Care and Use of Laboratory Animals (NIH Publications No. 80–23, revised 1996) and approved by the institution of Academy of Military Medical Sciences. All efforts were made to minimize animal suffering and reduce the number of animals used in the experiments.

### Preparation of the chronically stressed rats

The CUS model was performed as described previously with some modifications^24^. Except for control, the animals were subjected to the following stressors (from Mondays to Saturadays, except Sundays): (1) low-intensity stroboscopic illumination (80 flashes/min); (2) overnight illumination; (3) soiled cage (180 mL water in 90 g sawdust bedding); (4) food or water deprivation (24 h); (5) forced swimming (5 min at 10 °C); (6) 45° cage tilt; (7) white noise (approx. 110 dB); (8) restraint (2 h); and (9) tail pinch (2 min). All of the stressors were applied randomly and continuously. The rats in the control group were left undisturbed in the home cages, except for the 14 h period of water deprivation prior to each sucrose test. The outline of design for CUS was shown in Table (Table 1).

**Table 1:** Chronic unpredictable stress schedule.

Groups	Condition			
	Week 1	Week 2	Week 3	Week 4
Monday	Soiled cage: 24 h	Restraint: 2 h	Restraint: 2 h	Force swimming: 5 min
Tuesday	Overnight stroboscopic: 12 h	Tail pinch: 1 min	Overnight stroboscopic: 12 h	Food derivation: 24 h
Wednesday	Force swimming: 5 min	Overnight stroboscopic: 12 h	Food derivation: 24 h	Overnight illumination: 12 h
Thursday	Tail pinch: 1 min	Overnight illumination: 12 h	Water deprivation: 24 h	Soiled cage: 24 h
Friday	White noise: 1 h	Cage tilt: 24 h	Overnight illumination: 12 h	White noise: 1 h
Saturday	Water deprivation: 24 h	Water deprivation: 24 h	Force swimming: 5 min	Overnight stroboscopic: 12 h

#### Behavioral paradigms

**Figure 1:**
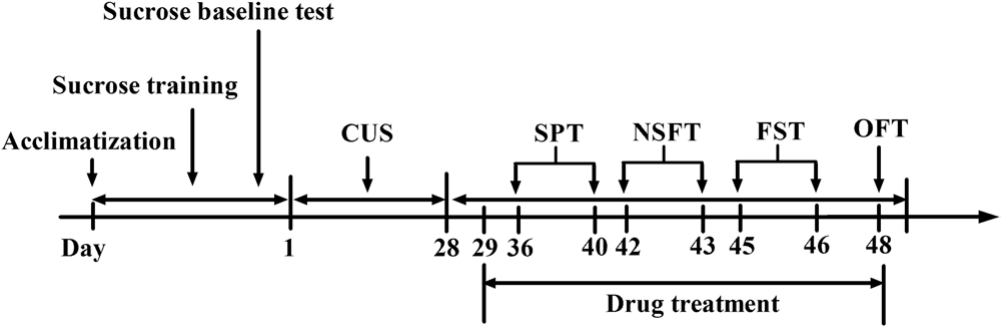
The outline of design for behavioral tests and the schedule of drug treatment.

After the acclimatization, rats were singly placed in cages and subjected to sucrose training and sucrose baseline test. The animals were divided into six statistically equivalent groups according to their sucrose intake in the final baseline test. After the 4-week CUS procedure, behavioral assessments including sucrose preference test (SPT) (from day 36 to 40), novelty-suppressed feeding test (NSFT) (from day 42 to 43), forced swimming test (FST) (from day 45 to 46), and open field test (OFT) (day 48) were performed on different days. The drugs were given to the rats from day 29 to the end of the behavioral tests. All the behavioral tests were performed 1 h after drugs administration. Control animals received 0.9% normal saline. The schedule of behavioral tests and drug treatment was outlined in Fig. 1.

### SPT

SPT is widely applied to measure the depressive-like behavior and performed as described previously^24, 29, 30^. Rats were placed in individual cages to train to consume 1% (w/v) sucrose solution for 48 h without food and water supply. Following 16 h water deprivation, one bottle of 1% sucrose solution was replaced by water. The position of the sucrose and water bottles for the SPT was counterbalanced. The SPT was performed for 1 h. During the test, rats could select between two preweighed bottles, one with 1% sucrose solution and the other with tap water. The sucrose preference was calculated as sucrose intake/(sucrose intake + water intake) ×100%.

### NSFT

The NSFT provides a sensitive and reliable measure of depressive-like behavior and motivation level in animals which mimics the situation in human^31, 32^. The test was performed according to the literature with minor modifications^24^. Briefly, after fasting for 24 h, each rat was placed in the corner of the plastic box (76 × 76 × 46 cm) with several pallets placed in the center. The latency to begin eating within 5 min was recorded (defined as chewing or biting the pallet, instead of merely sniffing or toying with it). Moreover, home-cage food consumption in 5 min was immediately determined to assess effects of drugs on feeding drive.

### FST

FST is a well-established measurement for evaluating the effects of antidepressants and the assessment is highly reliable to predict the validity of antidepressants^33, 34^. The procedure comprised two sections (the pretest and the test) with identical apparatus and conditions (height 40 cm, diameter 20 cm, containing 25 cm of water maintained at 25 °C). During the pretest section, rats were forced to swim for 5 min. After 24 h, rats were placed in the same apparatus for 5 min and the section was designated as a test section. The duration of immobility during 5 min was measured.

### OFT

To evaluate whether the reversion of depressive-like behavior by puerarin is dependent on an affect on locomotor activity, we assessed the number of crossings, rears, and fecal pallets in rats. The test was performed based on the literature with minor adjustments^35^. Each rat was placed in the corner of a plastic box (dimensions: 76 × 76 × 46 cm) that the base was divided into 16 equal squares for the 5-min acclimation period. Following that, the number of crossings (with all four paws placed into a new square), rears (with both front paws raised from the floor), and fecal pallets was rated by the observers who were blind to the grouping for 5 min by camera. The OFT was cleaned with a 5% ethanol/water solution after each test to remove any confounding olfactory cues and dried thoroughly between sections.

### Neurosteroids measurement

Altered levels of progesterone and allopregnanolone have been implicated as one of the possible contributors to the development of depressive-like behavior^23, 36^. The levels of neurosteroids in the antidepressant-like activity of puerarin were evaluated by enzyme linked immunosorbent assay (ELISA). The brain tissues preparation was based on a literature^24^. The brains were removed and carefully dissected to remove the prefrontal cortex and hippocampus at the end of OFT in 24 h after the last drug treatment. The brain regions were extracted by 1 mL extraction buffer per 100 mg tissue and then homogenized in the ice-cold lysis buffer containing 137 mM NaCl, 1% NP40, 10% glycerol, 20 mM Tris-HCl (pH 8.0), 1 μg/mL leupeptin, 1 mM PMSF 10 μg/mL aprotinin, and 0.5 mM sodium vanadate. The tissue homogenate solutions were centrifuged at 10,000 g for 25 min at 4 °C, and then the supernatants were collected. Progesterone and allopregnanolone were quantified by Enzyme Immunoassay kit (Progesterone: No. ADI-900–011, 15.62–500 pg/mL, Enzo Life Sciences, USA; Allopregnanolone: No. E1963Ge, 31.2–2000 pg/mL, EIAab, China). Six samples in each group were used to determine optical density (OD) values at 450 nm in ELISA plate reader (Beckman, USA) and used for statistical analyzes.

### Levels of Cort, CRH and ACTH measurement

The blood was sampled and collected at the end of behavioral tests in 24 h after the last drug treatment. The samples were centrifuged (2000 g, 30 min) at 4 °C and stored at −80 °C until further analyses. The test was performed based on the previous literature^37^. Levels of Cort, CRH and ACTH in serum were determined by ELISA kits (Cort: No. KGE009, 0.1–25 ng/mL, R&D Systems, USA; CRH: No. DL-CRH-Ra, 12.35–1000 pg/mL, Dldevelop, China; ACTH: No. DL-ACTH-Ra, 12.35–1000 pg/mL, Dldevelop, China) according to the manufacturer’s instructions. A sample (or standard) and conjugate were added to each well, and the plate was incubated for 1 h at room temperature. After several washes and proper color development, the OD value was determined at 450 nm by an ELISA plate reader (Beckman, USA).

### The determination of monoamine neurotransmitter levels

To further explore neurochemical mechanisms involved in antidepressant-like effect of puerarin, the levels of prefrontal cortex, hippocampus monoamine neurotransmitters and their metabolites were detected by high performance liquid chromatography-electrochemical detection (HPLC-ECD) after behavioral assessments. The detection was performed as described previously^7, 28^. Also, the brain tissues were dissected at the end of behavioral tests in 24 h after the last drug treatment on ice by a binocular dissection microscope and homogenized in an ice-cold tissue lysis buffer containing 0.1 g/L of L-cysteine, 0.5 mM Na_2_EDTA and 0.4 M HClO_4_ (5.0 μL/mg). The samples were centrifuged at 12,000 × g for 30 min at 4 °C and then filtered through a 0.45 μm pore membrane. The sample or standard solution was injected into the reversed-phase SunFire™ C18 column (250 mm × 4.6 mm, 5 μm) (Model C-18, DIKMA Technologies Ltd., China). Separation was performed in an isocratic elution mode at a column temperature of 20 °C using a mobile phase containing 0.1 M sodium acetate buffer (pH3.7) with 85 mM citric acid, 15% methanol, 0.9 mM sodium octanesulfonate, and 0.2 mM Na_2_EDTA at a flow rate of 1.0 mL/min. The monoamine neurotransmitters and the metabolites (5-HT, 5-HIAA, DOPAC, DA, AD, HVA and NE) were presented as ng/g wet weight of tissue.

### The antidepressant- and anxiolytic-like effects of puerarin by acute treatment

To evaluate whether the reversion of depressive- and anxiogenic-like behavior is induced by puerarin acute treatment, the FST and elevated plus maze test (EPMT) were performed, respectively. The FST was conducted as above. The EPMT is one of the widely utilized assessments for evaluating anxiogenic behavior in rodents. The apparatus consisted of four branching arms (60 × 12 cm) with two open arms and two closed arms with dark walls (40 cm high). The arms were connected by a centre platform (12 × 12 cm), and the maze was 50 cm above the ground. Each rat was placed in the central platform facing the closed arms. Rats were scored as entering an open or closed arm when all four paws passed over the dividing line. The exposure during initial 5 min was taped with a video camera. Time and numbers of entries into open arms were obtained as anxiety indices by an investigator who was blind to treatment conditions of animals. The percentage of time spent in and the entries into the open arms was calculated by dividing the time spent in and the entries into the open arms by the total time spent in and the total arm entries into the both arms, respectively. Both behavioral tests were performed 1 h after drug acute administration. Control animals received 0.9% normal saline.

### Statistical analysis

Statistical analysis was performed by GraphPad Prism 5.0 (GraphPad Software Inc., San Diego, CA). All data were presented as the mean ± S.E.M. The statistical significance of experimental observations was determined by one-way analysis of variance (ANOVA) followed by Bonferroni’s multiple comparison tests, as indicated in the results section. Differences in body weight gain were analyzed by two-way repeated measures ANOVA. For all tests, the level of statistical significance was set at *p* < 0.05.

## Results

### Effects of puerarin on the CUS-induced behavioral deficits in SPT

**Figure 2:**
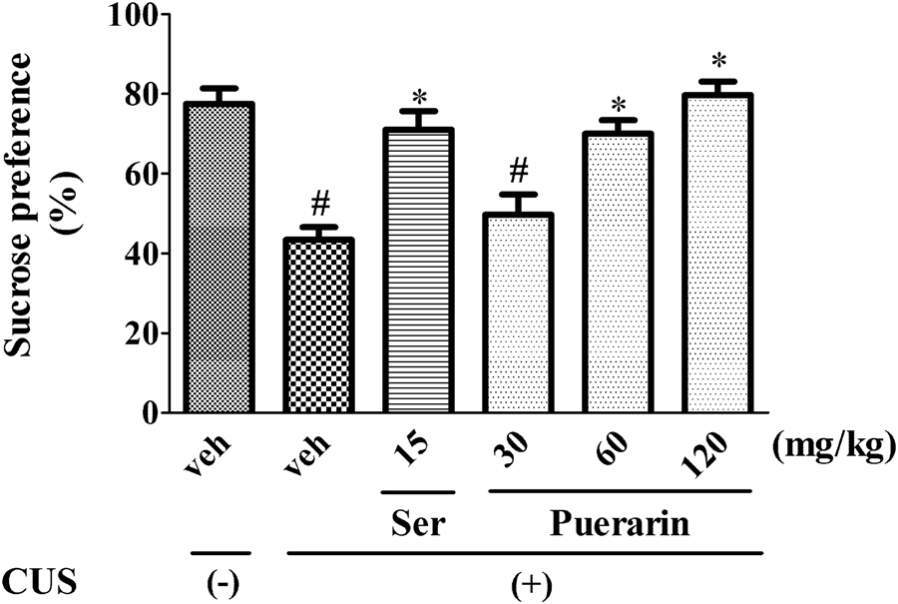
Puerarin attenuated the CUS-induced behavioral deficits in SPT. ^#^ *p* < 0.05 vs. vehicle-treated CUS (−) group; *p < 0.05, **p < 0.01 vs. vehicle-treated CUS (+) group (n = 10 per group).

The effects of puerarin on CUS rats in SPT were shown in Fig. 2. Sucrose preference was significantly decreased in CUS rats. Consistent with Ser (15 mg/kg, i.g), puerarin (60 and 120 mg/kg, i.g) reversed the decreased sucrose preference (F (5,54) = 14.25, *p* = 0.0000; Fig. 2) in CUS rats. The results indicated that puerarin ameliorated the CUS-induced behavioral deficits in SPT.

### Effects of puerarin on the CUS-induced behavioral deficits in NSFT

**Figure 3:**
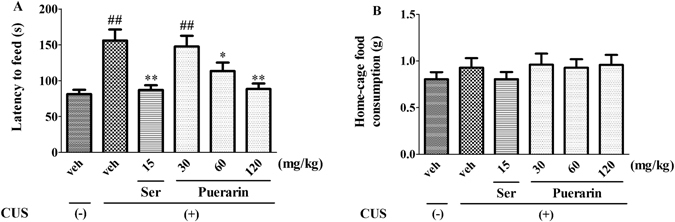
Puerarin attenuated the CUS-induced behavioral deficits in NSFT. The latency to feed was increased by CUS and reversed by puerarin. ^##^ *p* < 0.01 vs. vehicle-treated CUS (−) group; *p < 0.05, **p < 0.01 vs. vehicle-treated CUS (+) group (n = 10 per group).

As shown in Fig. 3, the latency to feed was increased significantly in CUS rats. Consistent with Ser (15 mg/kg, i.g), puerarin (60 and 120 mg/kg, i.g) blocked the increase of latency to feed (F (5,54) = 8.889, *p* = 0.0131; Fig. 3A). Moreover, no differences of in home-cage food consumption were observed among the groups (F (5,54) = 0.5905, *p* = 0.7072; Fig. 3B). The results indicated that repeated treatment of puerarin ameliorated the CUS-induced behavioral deficits in NSFT.

### Effects of puerarin on the CUS-induced behavioral deficits in FST

**Figure 4:**
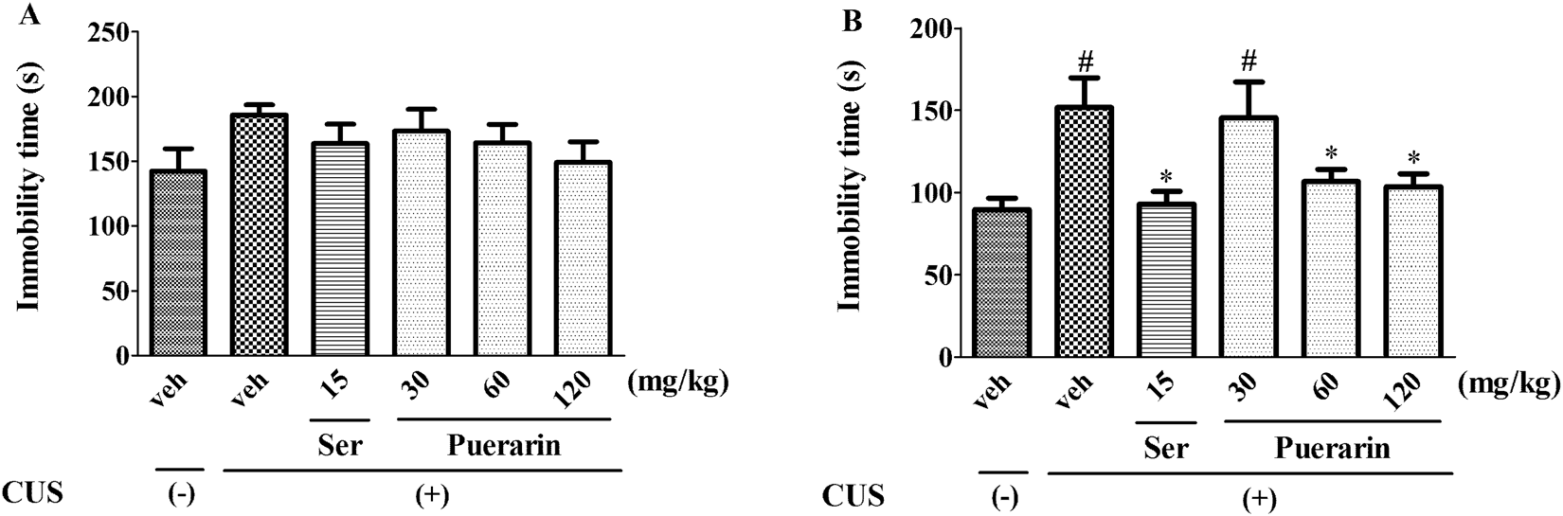
Puerarin attenuated the CUS-induced behavioral deficits in FST. ^#^ *p* < 0.05 vs. vehicle-treated CUS (−) group; ^*^ *p* < 0.05, ^**^ *p* < 0.01 vs. vehicle-treated CUS (+) group (n = 10 per group).

The effects of puerarin in FST were shown in Fig. 4. There was no significant effect on the immobility time (F (5,54) = 1.145, *p* = 0.3483; Fig. 4A) in pretest duration among groups. However, the immobility time was increased significantly in CUS rats in test section. Similar to Ser (15 mg/kg, i.g), puerarin (60 and 120 mg/kg, i.g) produced the antidepressant-like effects, as evidenced by the decreased immobility time (F (5,54) = 4.253, *p* = 0.0025; Fig. 4B). The results indicated that repeated treatment of puerarin ameliorated the CUS-induced behavioral deficits in FST.

### Effects of puerarin on the locomotor activity and body weight gain in rats

**Figure 5:**
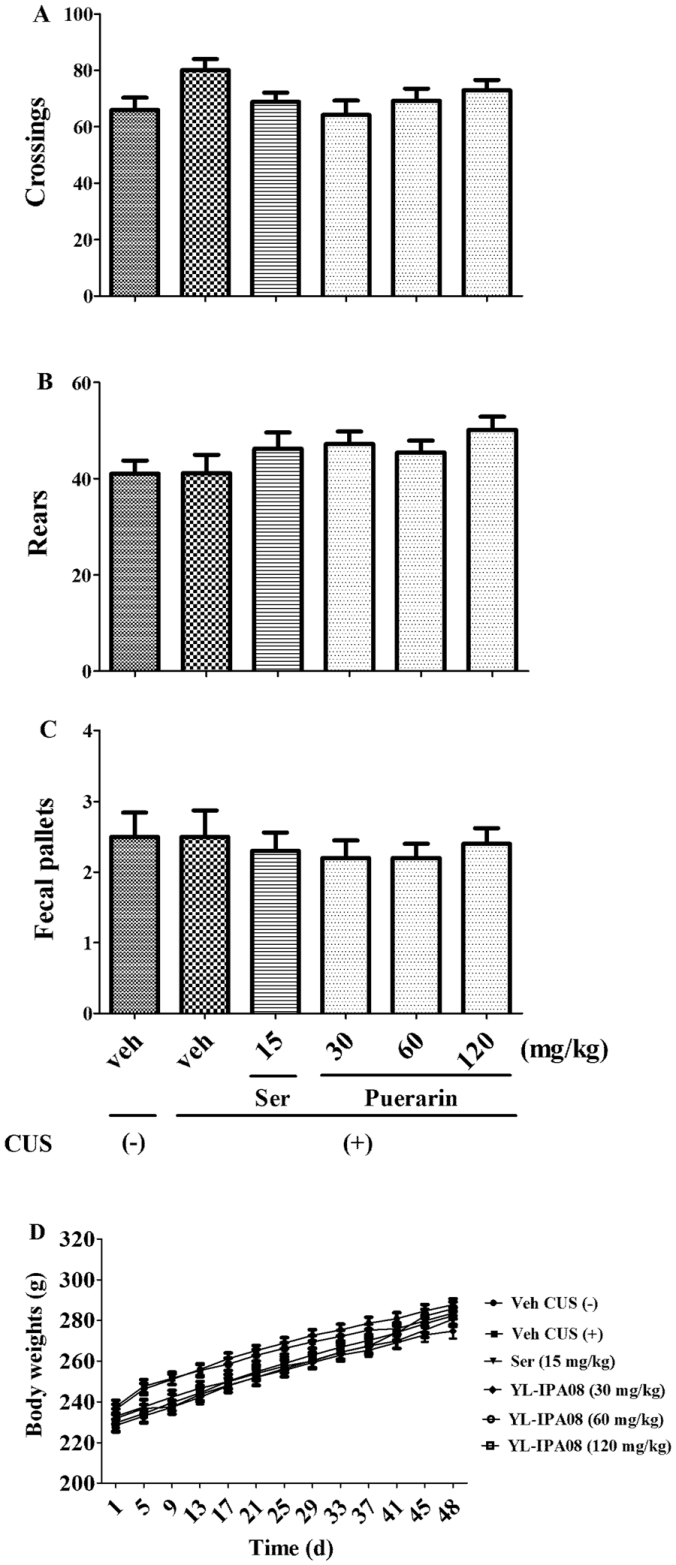
The effects of puerarin on locomotor activity. None of the treatments altered the number of line crossings (**A**), rears (**B**), and fecal pallets (**C**) in OFT. The body weights (**D**) were not altered by puerarin (n = 10 per group).

The effects of puerarin on locomotor activity were shown in Fig. 5. There was no significant effect on the number of line crossings (F (5,54) = 1.922, *p = *0.1058, Fig. 5A), rears (F (5,54) = 1.409, *p* = 0.2359, Fig. 5B), or fecal pallets (F (5,54) = 0.2403, *p* = 0.9429, Fig. 5C) among groups. In addition, body weight gain (F(5,54) = 1.389, *p* = 0.2387; Fig. 5D) was monitored throughout the drug treatment duration. In the puerarin-treated group, body weights was increased with time in a manner similar to that of the vehicle-treated group. These results indicated that neither puerarin treatment nor CUS modeling affected locomotor activity or body weight gain in rats.

### Effects of puerarin on the neurosteroid levels in CUS rats

**Figure 6:**
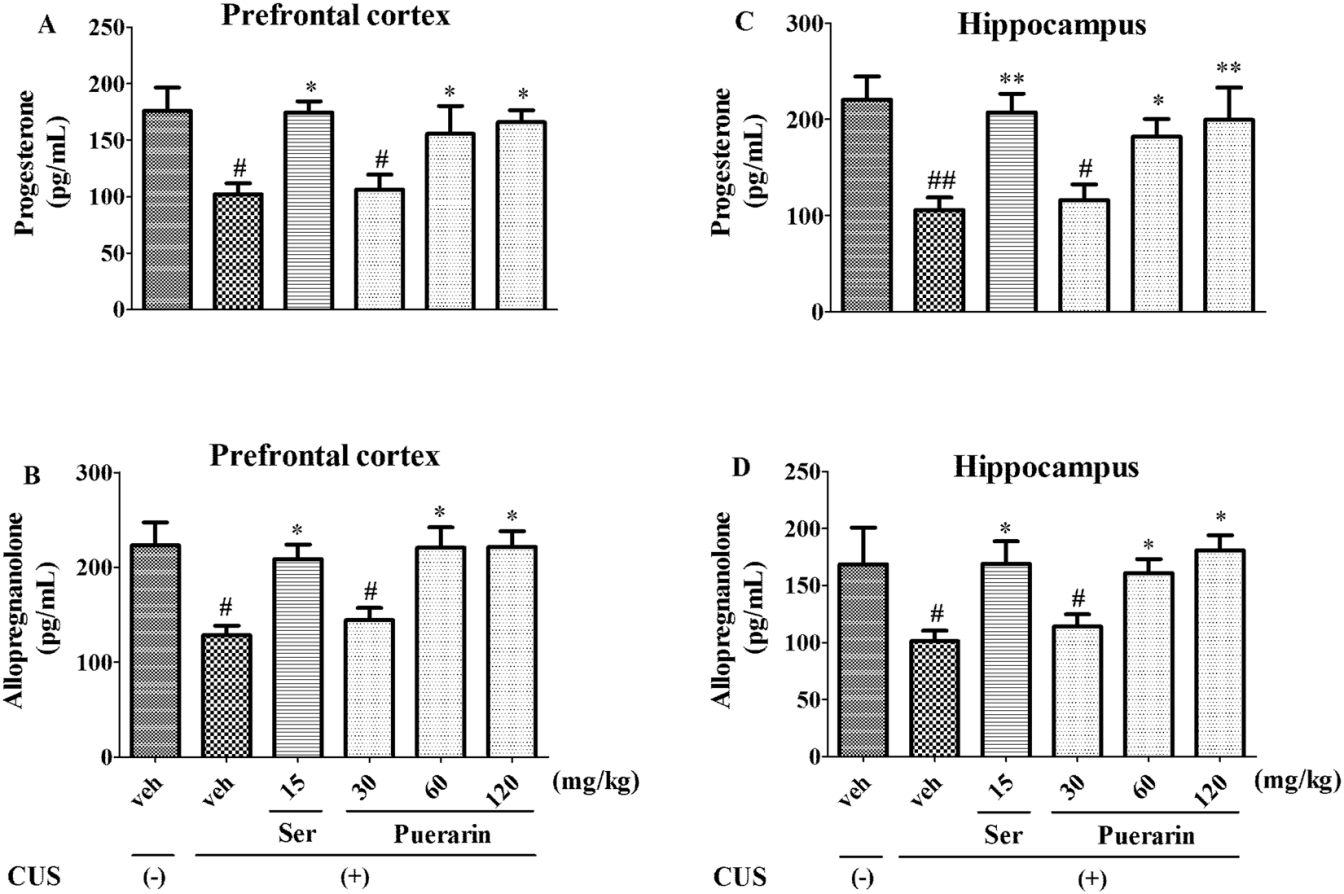
The effects of puerarin on levels of progesterone and allopregnanolone in the prefrontal cortex (**A**,**C**) and hippocampus (**B**,**D**), respectively. ^#^ *p* < 0.05, ^##^ *p* < 0.01 vs. vehicle-treated CUS (−) group; ^*^ *p* < 0.05, ^**^ *p* < 0.01 vs. vehicle-treated CUS (+) group (n = 6 per group).

The effects of puerarin on the levels of neurosteroids in CUS rats were shown in Fig. 6. After CUS exposure, levels of progesterone and allopregnanolone in the prefrontal cortex and hippocampus were significantly decreased, respectively. Similar to Ser (15 mg/kg, i.g.), both decreased levels of neurosteroids were significantly reversed by puerarin (60 and 120 mg/kg, i.g.) in the prefrontal cortex (F (5,30) = 4.510, *p* = 0.0035, for progesterone, Fig. 6A; F (5,30) = 6.102, *p* = 0.0005, for allopregnanolone, Fig. 6B) and hippocampus (F (5,30) = 4.993, *p* = 0.0019, for progesterone, Fig. 6C; F (5,30) = 3.377, *p* = 0.0154, for allopregnanolone, Fig. 6D), respectively. These results indicated that puerarin attenuated the CUS-induced behavioral deficits were associated with the biosynthesis of progesterone and allopregnanolone in brain.

### Effects of puerarin on CUS-induced HPA axis changes

**Figure 7:**
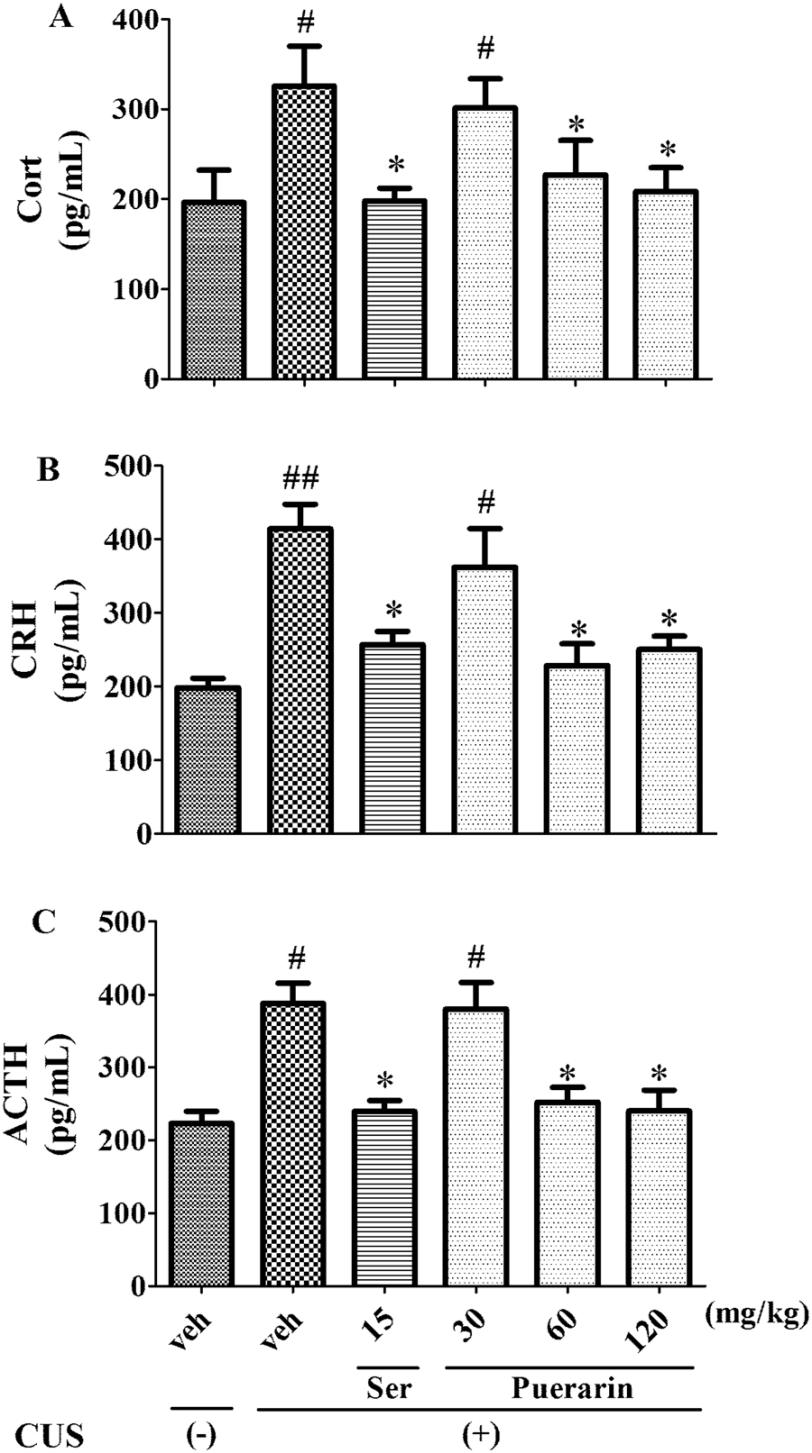
The effects of puerarin on Cort (**A**), CRH (**B**), ACTH (**C**) in serum. ^#^ *p* < 0.05, ^##^ *p* < 0.01 vs. vehicle-treated CUS (−) group; *p < 0.05, **p < 0.01 vs. vehicle-treated CUS (+) group (n = 6 per group).

The effects of puerarin on Cort, CRH and ACTH levels in rats were shown in Fig. 7. Following CUS exposure, levels of Cort (F (5,30) = 2.882, *p* = 0.0306; Fig. 7A), CRH (F (5,30) = 7.639, *p* = 0.0000; Fig. 7B) and ACTH (F (5,30) = 8.872, *p* = 0.0000; Fig. 7C) in serum were significantly increased. In accordance with Ser (15 mg/kg, i.p), these effects were significantly reversed by treatment with puerarin (60 and 120 mg/kg, i.g), respectively. These results indicated that the effects of repeated puerarin treatment on CUS-induced behavioral deficits were associated with decreased HPA stress hormone (Cort, CRH and ACTH) levels.

### Effects of puerarin on the monoamine neurotransmitter levels in CUS rats

The effects of puerarin on the levels of monoamine neurotransmitters in rats were shown in Table (Tables 2 and 3). After CUS exposure, levels of 5-HT and 5-HIAA in the prefrontal cortex and hippocampus were significantly decreased, respectively. Similar to Ser (15 mg/kg, i.g.), the decreased levels of 5-HT (F (5,30) = 3.538, *p* = 0.0124, for prefrontal cortex, Table 2; F (5,30) = 4.778, *p* = 0.0025, for hippocampus, Table 3) and 5-HIAA (F (5,30) = 2.919, *p* = 0.0290, for prefrontal cortex, Table 2; F (5,30) = 4.742, *p* = 0.0026, for hippocampus, Table 3) were significantly reversed by puerarin (60 and 120 mg/kg, i.g.), respectively.

**Table 2:** The effects of puerarin on prefrontal cortex monoamine neurotransmitter levels in CUS rats.

Groups	5-HT	5-HIAA	NE	AD	HVA	DA	DOPAC
CUS (−)	194.5 ± 26.15	147.0 ± 23.24	118.0 ± 12.12	153.7 ± 20.57	47.83 ± 4.408	43.50 ± 3.888	51.50 ± 6.893
CUS (+)	85.83 ± 9.325^##^	83.50 ± 9.929^#^	115.2 ± 14.47	165.0 ± 22.84	49.00 ± 6.787	51.83 ± 4.840	59.33 ± 5.200
Ser 15 mg/kg, i.g	163.0 ± 29.14*	151.8 ± 22.81*	130.5 ± 17.84	162.0 ± 22.89	60.50 ± 9.280	56.33 ± 6.238	52.67 ± 8.531
puerarin 30 mg/kg, i.g	126.5 ± 18.30	113.5 ± 11.30	138.2 ± 16.45	186.3 ± 21.84	60.33 ± 7.953	54.67 ± 7.491	53.33 ± 5.512
puerarin 60 mg/kg, i.g	181.5 ± 21.61*	149.2 ± 10.94*	134.5 ± 24.30	180.0 ± 25.41	50.50 ± 8.782	55.17 ± 6.740	52.83 ± 4.722
puerarin 120 mg/kg, i.g	164.5 ± 16.87*	158.5 ± 18.88*	125.2 ± 10.76	148.7 ± 14.97	59.33 ± 7.297	51.83 ± 5.902	55.17 ± 4.578

^#^ *p* < 0.05, ^##^ *p* < 0.01 vs. vehicle-treated CUS (−) group; *p < 0.05 vs. vehicle-treated CUS (+) group (n = 6).

**Table 3:** The effects of puerarin on hippocampal monoamine neurotransmitter levels in CUS rats.

Groups	5-HT	5-HIAA	NE	AD	HVA	DA	DOPAC
CUS (−)	205.5 ± 33.45	145.7 ± 25.66	140.7 ± 24.38	172.7 ± 24.49	47.17 ± 6.358	36.50 ± 5.602	49.83 ± 5.449
CUS (+)	99.17 ± 12.04^##^	58.83 ± 8.328^##^	123.7 ± 16.95	190.0 ± 24.24	44.33 ± 9.054	37.00 ± 6.282	45.50 ± 8.269
Ser 15 mg/kg, i.g	175.0 ± 19.23**	141.8 ± 17.66**	150.3 ± 12.39	193.5 ± 18.25	51.33 ± 6.136	30.50 ± 4.890	39.50 ± 4.904
puerarin 30 mg/kg, i.g	104.2 ± 6.194	92.33 ± 13.44	150.8 ± 20.49	176.5 ± 27.14	52.17 ± 8.856	33.67 ± 6.998	48.17 ± 7.748
puerarin 60 mg/kg, i.g	168.2 ± 12.62*	128.0 ± 24.85*	121.3 ± 15.81	205.8 ± 33.74	48.33 ± 8.617	39.33 ± 4.951	45.17 ± 4.020
puerarin 120 mg/kg, i.g	201.3 ± 30.03**	151.0 ± 28.28**	126.7 ± 21.50	190.5 ± 52.26	44.00 ± 4.389	38.17 ± 4.881	44.00 ± 5.342

^##^ *p* < 0.01 vs. vehicle-treated CUS (−) group; ^*^ *p* < 0.05, ^**^ *p* < 0.01 vs. vehicle-treated CUS (+) group (n = 6).

However, NE (F (5,30) = 0.3034, *p* = 0.9069, for prefrontal cortex, Table 2; F (5,30) = 0.4986, *p* = 0.7748, for hippocampus, Table 3), AD (F (5,30) = 0.4599, *p* = 0.8028, for prefrontal cortex, Table 2; F (5,30) = 0.1421, *p* = 0.9809, for hippocampus, Table 3), HVA (F (5,30) = 0.6397, *p* = 0.6711, for prefrontal cortex, Table 2; F (5,30) = 0.2120, *p* = 0.2120, for hippocampus, Table 3), DA (F (5,30) = 0.6057, *p* = 0.6960, for prefrontal cortex, Table 2; F (5,30) = 0.3288, *p* = 0.8916, for hippocampus, Table 3), DOPAC (F (5,30) = 0.2146, *p* = 0.9536, for prefrontal cortex, Table 2; F (5,30) = 0.3393, *p* = 0.8850, for hippocampus, Table 3) in both brain regions were not significantly affected by CUS and puerarin. These results indicated that the effects of repeated puerarin treatment on CUS-induced behavioral deficits were associated with the normalized levels of 5-HT and 5-HIAA in the prefrontal cortex and hippocampus.

### The antidepressant- and anxiolytic- like effects of acute puerarin treatment in rats

**Figure 8:**
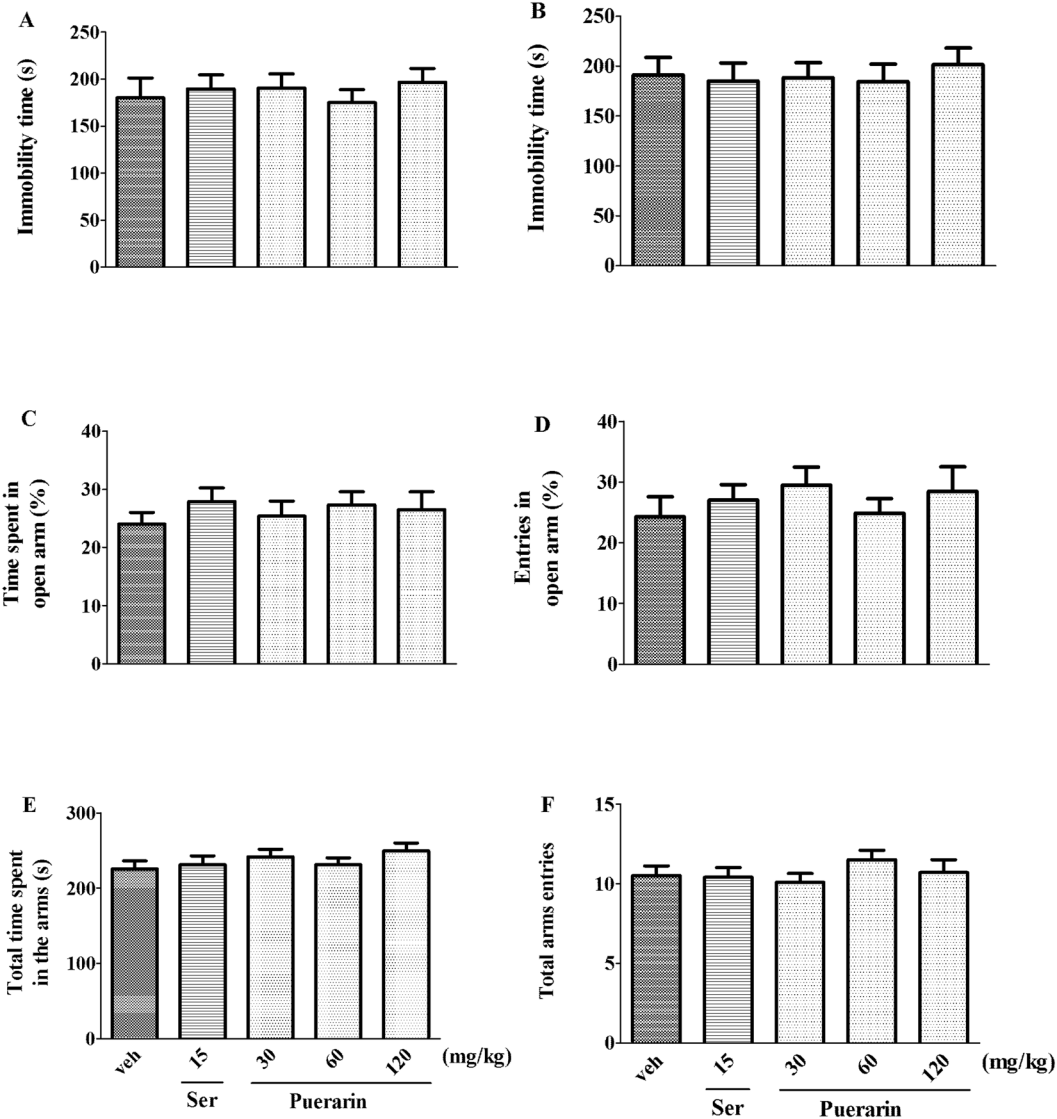
The acute effects of puerarin on FST and EPMT. In FST, there was no significant effect on the immobility time in pretest (**A**) and test (**B**) duration among groups. Similar to FST (15 mg/kg, i.g), both drugs did not induce the anxiolytic-like effects in EPMT, as evidenced by that the percentage of the time (**C**)/entries (**D**) into open arms and total time (**E**)/entries (**F**) in arms among groups were not different. (n = 10 per group).

The antidepressant- and anxiolytic- like effects of acute puerarin treatment in rats were shown in Fig. 8. In FST, there was no significant effect on the immobility time in pretest (F (4,45) = 0.3010, *p* = 0.8758; Fig. 8A) and test (F (4,45) = 0.1689, *p* = 0.9531; Fig. 8B) duration among groups. Similar to FST (15 mg/kg, i.g), both Ser and puerarin did not produce the anxiolytic-like effects in EPMT, as evidenced by that the percentage of the time (F (4,45) = 0.3949, *p* = 0.8112; Fig. 8C)/entries (F (4,45) = 0.5266, *p* = 0.7167; Fig. 8D) into open arms and total time (F (4,45) = 0.8678, *p* = 0.4906; Fig. 8E)/entries (F (4,45) = 0.6711, *p = *0.6154; Fig. 8F) in arms among groups were not different. The results indicated that acute puerarin treatment did not ameliorate the antidepressant- and anxiolytic- like effects in FST and NSFT, respectively.

## Discussion

In the present study, we preliminarily evaluate the effects of puerarin on CUS-induced behavioral deficits via the CUS model and its possible mechanism. The CUS-induced behavioral deficits were significantly ameliorated by puerarin without affecting locomotor activity in rats. In addition, together with the results of neurosteroids biosynthesis, serotonergic system and hormones of HPA axis, we found that the effects of repeated puerarin treatment on CUS-induced behavioral deficits were associated with biosynthesis of neurosteroids, normalization of serotonergic system and preventing HPA axis dysfunction.

Depressive-like behavior is a serious mental health problem associated with psychiatric morbidity and has been increased worldwide from past several decades^1, 2^. The CUS model mimics the depressive-like symptoms and is widely used in the preclinical antidepressants evaluation which offers a closer resemblance to chronic stress^24, 38^. CUS induced behavioral changes that resemble clinical depressive-like behavior, such as reduced sucrose intake and responsiveness to rewarding stimuli^24, 39^. In present study, CUS induced a significant reduction of sucrose preference in SPT (Fig. 2) and the increased immobility time in FST (Fig. 4B), two indicators of the core symptoms of depressive-like behavior. Furthermore, it has been documented that the CUS model may also induce anxiogenic-like symptoms, which is evidenced by the increased latency to feed (Fig. 3). The NSFT is initially used to examine the effects of anxiolytic agents, and also sensitive to chronic treatment with antidepressants^40, 41^. These findings were further supported by the successful experimental protocol of the CUS procedure.

The CUS-induced behavioral parameters can be reversed by the chronic administration of antidepressants^7, 24^. Our results showed that similar to Ser (15 mg/kg i.g.), puerarin (60 and 120 mg/kg i.g.) reversed the decreased sucrose preference to normal levels in SPT (Fig. 2) and the increased immobility time in FST (Fig. 4B). Moreover, the increased latency to feed in NSFT was blocked by puerarin (60 and 120 mg/kg i.g.) (Fig. 3A) without affecting home-cage food consumption (Fig. 3B), suggesting that puerarin attenuated the CUS-induced behavioral deficits in rats. The effective doses of puerarin (60 and 120 mg/kg i.g.) were almost confirmed among SPT (Fig. 2), FST (Fig. 4B) and NSFT (Fig. 3A) and in line with depressive-like behavioral deficits that were reversed by puerarin with the similar doses^16^. Moreover, the OFT was also performed to evaluate whether the reversion of chronic stress behavioral deficits by puerarin was dependent on locomotor activity. Consistent with the previous findings and Ser (15 mg/kg i.g.)^28^, the present study also showed that the locomotor activity (e.g line crossings, rears and fecal pallets) was not affected by puerarin in OFT (Fig. 5). In addition, these findings were also consistent with our observations found that the anxiolytic activities of puerarin determined by EPMT which the total time (Fig. 8E) and entries (Fig. 8F) in arms were not significantly altered by puerarin. The data indicated that the effects of puerarin on the CUS-induced behavioral deficits were not mediated by affecting the locomotor activity in rats.

Ser was delivered 1 h before behavioral experiments and exerted the anti-stress activities with its chronic treatment^27, 42^. Therefore, puerarin had the similar the treatment schedule with Ser and showed that both drugs elicited the antidepressant-like effects via behavioral tests in present study. The study showed that the behavioral experiments were performed after the drug treatment at least 1 week (chronic/subchronic treatment). Moreover, our studies also found that the single treatment of puerarin 1 h before the behavioral experiments in rodents did not produce the anxiolytic- and antidepressant- like effects (Fig. 8). The findings were supported by the antidepressant-like effects of puerarin with repeated treatment^16^. Thus, it indicated that the effects of puerarin might be induced by its chronic treatment effect.

It was reported that dysfunction of the prefrontal cortex or hippocampus is implicated in the pathogenesis of depressive-like behavior^34, 43^. Both brain regions play an important role in fear conditioning, emotional processing and explicit memory that are relevant to anxiogenic- and depressive-like behavior^43–45^. Thus, to confirm the role of neurosteroids in the effects of puerarin on the CUS-induced behavioral deficits, we then measured levels of endogenous neurosteroids and monoamine neurotransmitters in the above brain regions.

Although the pathological factor of depressive-like behavior have been widely researched, the exact factors involved are still not discovered. More evidences demonstrate that the biosynthesis of neurosteroids (e.g. progesterone and allopregnanolone) has been implicated as one of the possible contributors in the development of depressive-like behavior^46, 47^. The present findings showed that similar to Ser, both decreased levels of neurosteroids were significantly reversed by puerarin in the prefrontal cortex (Fig. 6A and B) and hippocampus (Fig. 6C and D), respectively. The results indicated that the effects of puerarin on the CUS-induced behavioral deficits were associated with the biosynthesis of progesterone and allopregnanolone in brain. Our present study was also confirmed by the finding that altered levels of progesterone affected the levels of metabolite steroids, such as allopregnanolone, and progesterone withdrawal in animals also dramatically reduces levels of allopregnanolone in brain^48^.

Progesterone serves as the main precursor molecule for 3β-pregnane neuroactive steroids which possessed antidepressant-like activities^49^. The beneficial effect of progesterone may come after its conversion to allopregnanolone and through that metabolite’s agonistic action at GABA (γ-aminobutyric acid) A receptors^47^. Allopregnanolone is the brain neurosteroid that acts GABAA receptors and is the selective positive endogenous modulator of the action of GABAA at GABAA receptors in brain^49, 50^. More clinical studies have shown that depressive-like behavior is closely associated with the biosynthesis of neurosteroids. For instance, decreased allopregnanolone in peripheral blood or cerebrospinal fluid (CSF) is found to relevant to MDD, anxiety disorders, premenstrual dysphoric disorders, negative symptoms in schizophrenia, or impulsive aggression^51^. These might be related to the effects of levels of allopregnanolone on the regulation of GABAA receptor function, which plays a role in the pathophysiology of depressive-like behavior. The GABAA agonist modulator interacted on the brain by changing the expression of GABAA receptor subunit to produce the neuroprotective effects on the depressive-like behavior^52^.

Moreover, the hyperactivity of the HPA axis, which is commonly seen in patients with depressive-like symptoms, is the most commonly observed neuroendocrine abnormality in MDD^53^. Here, we found that the increased levels of Cort (Fig. 7A), CRH (Fig. 7B) and ACTH (Fig. 7C) in serum of post-CUS rats. The findings were supported by that the increased levels of CRH, Cort and ACTH in menopause depressive ovariectomized rats under chronic unpredictable mild stress^54^. The results were also consistent with the previous study that the role of allopregnanolone as an endogenous negative regulator of HPA axis activity, and plasma Cort was elevated concomitantly with decreased levels of allopregnanolone in chronically stressed rats^55^. Interestingly, study also demonstrated that the hormones of HPA axis above in post-CUS rats were reversed by puerarin (Fig. 7), which indicated that the normalization of brain neurosteroid levels and the subsequent prevention HPA axis dysfunction may underlie the activities of puerarin on the CUS-induced behavioral deficits.

In mammals, the HPA axis and the monoamines system closely interact in central nervous system (CNS) (particularly in prefrontal cortex and hippocampus) and are greatly involved in stress-related disorders^56, 57^. Based on these evidences, the role of monoamines in the effects of puerarin on the CUS-induced behavioral deficits were evaluated. Our findings demonstrated after CUS exposure, levels of 5-HT and 5-HIAA in both brain regions were significantly decreased (Tables 2 and 3). The results were confirmed with decreased serotonin levels (e.g 5-HT and 5-HIAA) in a CUS animal model^7^. As the pathophysiological theory of depressive-like behavior, the monoamine hypothesis holds that lowered levels of 5-HT in the CNS are closely associated with depressive-like behavior^35^. However, similar to Ser, the decreased levels of 5-HT and 5-HIAA were significantly reversed by puerarin (Tables 2 and 3), indicating that the effects of puerarin on the CUS-induced behavioral deficits were associated with the normalized levels of 5-HT and 5-HIAA in brain. Substantial evidences indicated that depressive-like behavior was due to an absence of brain monoaminergic activity^7, 35^, and the levels of monoamine neurotransmitters (e.g 5-HT) in brain were increased in the experimental groups after the antidepressant treatments^58^.

In neurological study, reports showed that puerarin elicited the potential effects on attenuating memory and learning disorders, including Parkinson’s disease, Alzheimer’s disease, ischaemic stroke *et al*.^59–61^. However, not many studies on the antidepressant-like effect of puerarin and its possible mechanism. It is possible that puerarin normalizes the depressive-like behavior by other alternative mechanisms, such as activation of brain-derived neurotrophic factor (BDNF) and glutamate receptor. Recently, study showed puerarin ameliorates depressive-like behaviors and chronic pain in mice with SNI which markedly promoted the activation of CAMP-response element binding protein (CREB) pathway and induced BDNF expression^16^. BDNF plays an important role on neuronal survival, neurogenesis, differentiation of neurons and synapses, learning and memory^62^. Interestingly, BDNF was decreased in hippocampus and prefrontal cortex of patients with depressive-like symptoms and other psychiatric disorders^63^. Increased BDNF expression could largely improve depressive disorders^64^. Thus, BDNF was recently suggested as an important biomarker for successful treatment of depressive-like behaviors. Puerarin profoundly induced the phosphorylation of CREB and expression of BDNF^16^. Therefore, it is possible that puerarin promotes the cross-talks between BDNF-CREB signaling pathways.

Moreover, the glutamate receptors have also been examined as potential therapeutic targets for depressive-like behavior^65^. Puerarin ameliorated learning and memory deficits in mice through normalizing the glutamatergic/GABAergic system and causing synaptic structural modifications in the hippocampus^66^. Previous study showed that neuroprotective effect of puerarin was potentially mediated through the inhibition of glutamate-induced activation of mitochondrial-dependent signaling pathway and calmodulin-dependent protein kinase II (CaMKII)-dependent apoptosis signal-regulating kinase 1(ASK-1)/c-Jun N-terminal kinase (JNK)/p38 signaling pathway^67, 68^.

Taken together, the present study showed that puerarin exerted the ameliorative activities via the CUS-induced stress and various behavioral tests. The possible mechanism may be associated with biosynthesis of neurosteroids, normalization of serotonergic system and preventing HPA axis dysfunction, which may account for the molecular and cellular mechanism underlying the effects of puerarin on the CUS-induced behavioral deficits. Based on the previous and present study, it indicates that not only advance our knowledge of the theories of MDD, but also have clinical implications for puerarin that may serve as a novel compound for the therapeutics of depressive-like behavior. Although we preliminarily evaluated the effects of puerarin on CUS-induced behavioral deficits and its possible mechanism, the effect of chronic puerarin on behavior and molecular readouts was not fully performed. Thus, it is possible that partial activity may not be specific to CUS. More work should be conducted the chronic puerarin in rats to provide more evidences for the chronic activity of puerarin on CUS-induced behavioral deficits and molecular pathways/targets. In addition, the pharmacodynamics of puerarin in CNS towards the clinical translation are also needed.
